# Strain-induced control of a pillar cavity-GaAs single quantum dot photon source

**DOI:** 10.1038/s41598-019-55010-3

**Published:** 2019-12-06

**Authors:** Inah Yeo, Doukyun Kim, Il Ki Han, Jin Dong Song

**Affiliations:** 1 Dielectrics and Advanced Matter Physics Research Center, Pusan National University, Busan, 46241 Korea; 2 Nanophotonics Research Center, Korea Institute of Science and Technology, Seoul, 02792 Korea; 3 Post-Silicon Semiconductor Institute, Korea Institute of Science and Technology, Seoul, 02792 Korea

**Keywords:** Materials science, Nanoscience and technology, Optics and photonics

## Abstract

Herein, we present the calculated strain-induced control of single GaAs/AlGaAs quantum dots (QDs) integrated into semiconductor micropillar cavities. We show precise energy control of individual single GaAs QD excitons under multi-modal stress fields of tailored micropillar optomechanical resonators. Further, using a three-dimensional envelope-function model, we evaluated the quantum mechanical correction in the QD band structures depending on their geometrical shape asymmetries and, more interestingly, on the practical degree of Al interdiffusion. Our theoretical calculations provide the practical quantum error margins, obtained by evaluating Al-interdiffused QDs that were engineered through a front-edge droplet epitaxy technique, for tuning engineered QD single-photon sources, facilitating a scalable on-chip integration of QD entangled photons.

## Introduction

Single-photon sources (SPSs) based on semiconductor quantum dots (QDs) have been developed for high-performance quantum computation and technologies^1–3^. Conventional self-assembled QDs exhibit near-perfect quantum emission properties^4–6^, making them highly promising for use in quantum-information processing^7–11^. However, despite the high potential of solid-state SPSs, the deterministic control (e.g., compositional profiles and strain-related defects) of self-assembled QDs is rendered difficult by the strain-driven self-assembly process accompanied by lattice mismatches in the epilayer/substrate heterostructures. To alleviate this problem, researchers have fabricated strain-free GaAs QDs via droplet epitaxy^12–18^ or by filling self-assembled nanoholes^19–23^. Potentially, strain-free QDs can be engineered to specific sizes and shapes, realizing high-fidelity entangled photon sources^24–26^. Moreover, the individual energies of single two-level systems can be effectively controlled by applying stress to the QD SPSs^26–31^.

Our approach to single-QD control is based on a micropillar cavity optomechanical resonator embedded with strain-free GaAs/AlGaAs QDs. We exhibit the stress-induced control of the individual QD exciton dynamics, which would permit emitter-emitter and cavity-emitter resonant couplings. Additionally, we theoretically evaluate the quantum mechanical corrections of the QD band structures using a three-dimensional (3D) envelope-function method. We provide the practical limits on the tuning/position precision, which we ascribe to the QD shape asymmetry and individual composition profiles. The hybrid tuning scheme can conceptually create quantum bits in QDs^30–36^, The QD-cavity structures (vertical-cavity surface-emitting laser or VCSEL-type) are considered to be the most promising platform for efficient light collection^4,5,37^. As some hybrid quantum systems have been implemented in the past^38–40^, the optomechanical techniques are critical for improving the scalability of QD SPSs in these systems.

## Results and Discussions

**Figure 1:**
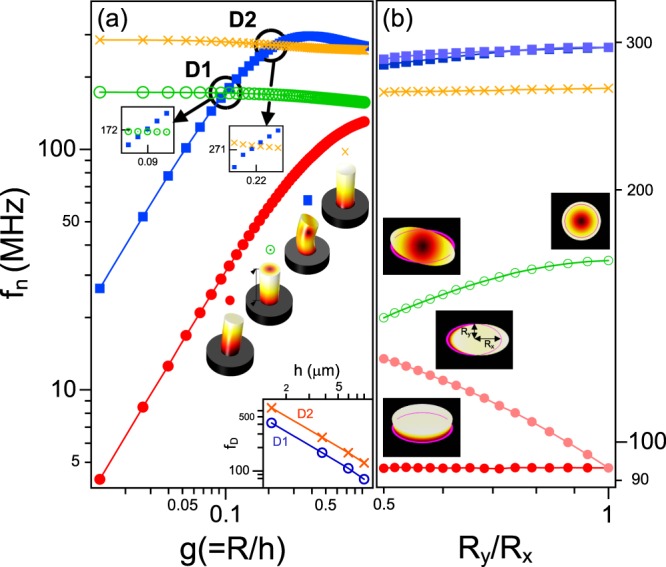
(**a**) Size- and (**b**) shape-dependent resonance frequencies of a GaAs/AlGaAs Bragg micropillar cavity as functions of the pillar aspect ratio *g *(= *R*/*h*) between the radius, *R*, and height, *h*, and pillar axial ratio *R*_*y*_/*R*_*x*_ at *g* ~ 0.4. To construct a strain-free GaAs/Al_0.3_Ga_0.7_As QDs in a cavity system, we modeled an Al_0.3_Ga_0.7_As *λ *(= 241 nm)-cavity enclosed by (*λ*/4 = 50.5 nm, *λ*/4 = 60.7 nm) GaAs/Al_0.9_Ga_0.1_As Bragg reflectors (17 and 15 periods in the bottom and top reflectors, respectively). The micropillar contains GaAs QDs in the center of the Al_0.3_Ga_0.7_As *λ*-cavity. The cavity’s resonant mode is optimized to the conventional QD exciton energy, i.e., 1.653 eV. The red circles, blue squares, green circled dots, and yellow crosses in the figure indicate the first and second flexural modes, and the radial and longitudinal breathing modes, respectively. The multimode deformation profiles have been magnified for clarity. The inset demonstrates the frequency shift, *f*_*D*_, of the degenerate points D1 and D2 as a function of the height *h*(μm). For the four DBR pillars, the numbers of the bottom (top) reflectors are 7 (5), 17 (15), 27 (25), and 37 (35).

Adjusting the nano- or micro-scale distributed Bragg reflector (DBR) cavity of a singly clamped pillar offers several degrees of freedom with completely different natures. Figure 1 shows how the frequencies of the four characteristic oscillation eigenmodes in a standard micropillar cavity depend on the pillar aspect ratio *g* (defined as *R*/*h*, where *R* and *h* are the radius and height of the pillar, respectively). These results were obtained via a finite element simulation. As the pillar widened relative to its height (*g* < 0.3), the eigenfrequencies *f*_*n*_ of the first two flexural modes increased such that ${f}_{n}={\beta }_{n}^{2}/(2\pi {h}^{2})\sqrt{YI/\rho A}$ ^41^. Here, the first two eigenvalues of the vibration modes correspond to *β*_1_ = 1.875 and *β*_2_ = 4.694^42^. For Al_*x*_Ga_1−*x*_ As epitaxial structures, the alloy-composition-dependent Young’s modulus *Y* and mass density *ρ* correspond to (85.3 − 1.8*x*) [MPa] and (5320 − 1560*x*) [kg/m^3^], respectively^43^. In addition, *I* = *πR*^4^/4 is the area moment of inertia and *A* is the cross-sectional area. While the longitudinal eigenfrequencies were almost independent of *g*, the hybrid mode frequencies degenerated at two points within a relative frequency splitting (Δ*f*/*f*) of 10^−3^, which are labeled D1 and D2 (with *g*_D1_ ~ 0.1 and *g*_D2_ ~ 0.2, respectively) in Fig. 1. The hybrid resonances with higher-order modes can be set in a strong-coupling regime^44,45^, controlling the resonant dynamics via the coherent exchange of phonons^46–48^. In our DBR-based SPS prototype, the degenerate eigenfrequencies of the two hybrid modes were tunable from several tens of MHz to ~650 MHz.

Controlling the pillar ellipticity adds additional degrees of freedom, leading to asymmetric mechanical polarization distributions. The semi-major and semi-minor radii of the elliptic pillar resonators are denoted as *R*_*x*_ and *R*_*y*_, respectively. As the ellipse’s axial ratio *R*_*y*_/*R*_*x*_ reduced toward 0.5, the fundamental oscillation frequency of the major axis increased with a relative frequency splitting of 0.4. Elliptical VCSEL resonators with low axial ratios developed torsional breathing modes (green open circles in Fig. 1(b)). The longitudinal breathing characteristics were independent of the pillar ellipticity. In the second flexural mode, two separate elliptic modes appeared within 2% of the resonant frequencies.

**Figure 2:**
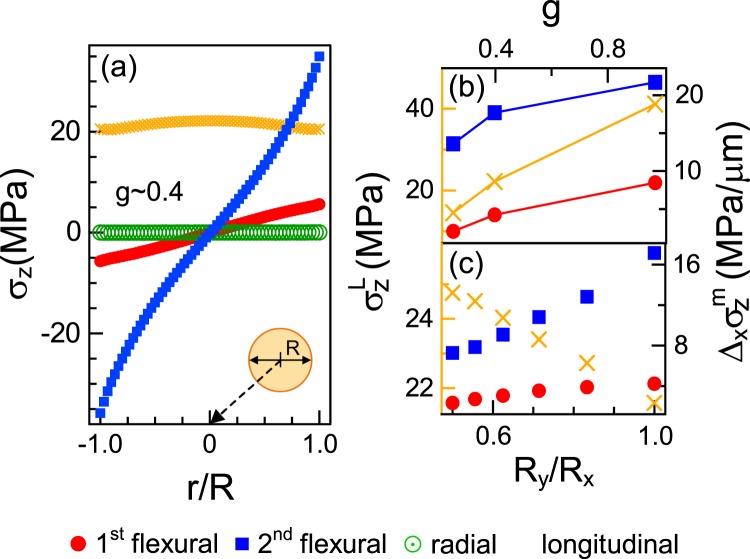
(**a**) Representative principal stresses *σ*_*z*_ of multiple modes acting on the QD plane in a cylindrical micropillar cavity resonator with *g* ~ 0.4. (**b**) Size and (**c**) shape dependencies of the maximum longitudinal stress ${\sigma }_{z}^{L}$ and the stress-field gradient $\Delta {\sigma }_{z}^{m}(|r/R|$ < 0.4) near the center. The left (yellow) and right axes correspond to the longitudinal uniaxial stress ${\sigma }_{z}^{L}$ and the cross-sectional stress-field gradient $\Delta {\sigma }_{z}^{m}$ of the flexural modes *m*, respectively.

These distinct multimode mechanical vibrations were accompanied by non-uniform strain fields in the pillar geometries. Figure 2(a) shows typical in-plane principal-stress components of the various modes applied on the QD embedded in the middle of the AlGaAs cavity layer inside the pillar. The stress components linearly varied by several tens of MPa. Owing to the quadratic distributions of stress in the flexural modes and the non-uniformity of the longitudinal stress, our investigation of the stress-field gradients ${\Delta }_{x}{{\rm{\sigma }}}_{{\rm{z}}}^{{\rm{m}}}$ is restricted in the ranges |*r*/*R*| < 0.4. The ${\Delta }_{x}{{\rm{\sigma }}}_{{\rm{z}}}^{{\rm{m}}}$ along the *x*-axis were determined as 4 and 17 MPa/μm for the first two flexural modes, respectively. We adjusted the aspect ratio of the VCSEL pillar from 0.2 to 0.9 at a fixed radius of 1.5 μm such that a high Purcell factor could be obtained^49^. Uniaxial stresses, ${\sigma }_{{z}}^{{\rm{L}}}$, of up to 40 MPa can be generated in the QD plane by longitudinally displacing the free-end of the cylindrical pillar by 1 nm (≈10^6^ *x*_zpa_) at *g* ~ 0.4 (see Fig. 2(b)). Here, *x*_zpa_ denotes the zero-point amplitude of the first flexural mode. In addition, we calculated the uniform in-plane mean stress profiles at 0 MPa and 22 MPa for the radial and longitudinal breathing modes, respectively. As Fig. 2(c) demonstrates, increasing the ellipticity decreased the axial stress gradient. The gradient of a highly elliptic cavity resonator (axial ratio 0.5) was almost 50% that of the symmetric flexural resonances. The (ellipticity-independent) longitudinal resonance simply increased the relative principal stress by 15%. Here, the sign of the applied stress depends on the vibrational phase.

At each resonance, an applied stress can change the exciton energy levels of the strain-free GaAs QDs embedded in a cylindrical VCSEL pillar resonator. A single crystal under uniaxial stress undergoes various deformation processes. First, a hydrostatic stress produces the isotropic crystal lattice distortion, shifting the QD conduction/valence band extrema to higher/lower energies, relative to the mechanical phase. Second, a shear stress lowers the symmetry of zinc-blend crystals, splitting their heavy- and light-hole valence states. Consequently, the bandgap energy between the heavy hole and conduction bands also shifts to a lower energy. Under an external (tensile) uniaxial stress, the reduced QD height modifies the quantum potential. Such a change in the confinement potential not only increases the electron–hole sub-band confinement energies, but also alters the strength of the electron–hole Coulomb interaction. Even small intrinsic strains in GaAs/AlGaAs QDs can significantly alter the quantum confinement parameters^15^.

**Figure 3:**
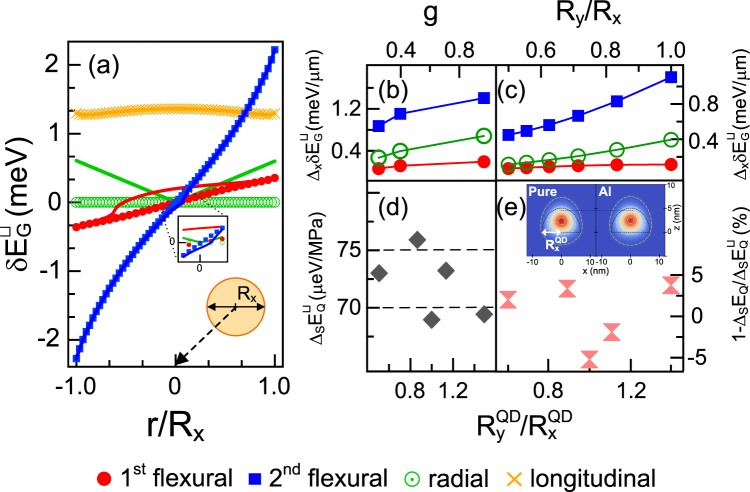
(**a**) Influence of stress on the QD excitonic bandgap energy $\delta {E}_{{\rm{G}}}^{\bigsqcup }$ in the middle layer of the *λ*-AlGaAs cavity in a cylindrical micropillar with *g* ~ 0.4. Effect of (**b**) pillar aspect ratio *g* and (**c**) ellipticity on the cross-sectional shift rate ${\Delta }_{x}\delta {E}_{{\rm{G}}}^{\bigsqcup }$ of the two flexural modes (red circles and blue squares) and the radial breathing mode (green open circles). (**d**) Stress-induced changes in the quantum confinement energy ${\Delta }_{{\rm{S}}}{E}_{{\rm{Q}}}^{\bigsqcup }$. As an example, we plot the sum of QD sub-band and Coulomb interaction energies as a function of the geometrical asymmetry ${R}_{y}^{{\rm{QD}}}/{R}_{x}^{{\rm{QD}}}$ of the QDs. Here, ${R}_{x(y)}^{{\rm{QD}}}$ denotes the major(minor) radius of a lens-shaped strain-free GaAs QD. (**e**) Effect of Al interdiffusion on the stress-induced quantum correction ${\Delta }_{{\rm{S}}}{E}_{{\rm{Q}}}^{\bigsqcup }$. The dependence of $1-{\Delta }_{{\rm{S}}}{E}_{{\rm{Q}}}^{{\rm{Al}}}/{\Delta }_{{\rm{S}}}{E}_{{\rm{Q}}}^{\bigsqcup }$ on the QD axial ratio is plotted as a percentage. The inset shows the probability density distribution |*ψ*(**r**)|^2^ of the lowest hole state localized in a pure QD (left) and an Al-interdiffused QD (right).

Figure 3(a) depicts the calculated shift in the QD excitonic bandgap energy $\delta {E}_{{\rm{G}}}^{\bigsqcup }$ under characteristic multimode strain fields (e.g., Fig. 2(a)). To exploit our system as an optomechanical oscillator for QD SPSs, we examined the magnitudes of the rates of change of ${\Delta }_{x}\delta {E}_{{\rm{G}}}^{\bigsqcup }$ in the core region |*r*/*R*| < 0.4. We tuned the cross-sectional rates of change to 0.2, 1.4, and 0.7 meV/μm, obtaining the first flexural mode, the second flexural mode, and the radial breathing mode at g ~ 0.9, respectively (Fig. 3(b)). In the longitudinal mode, we observed an almost uniform energy shift of the GaAs QDs embedded in the micropillar cavity. Notably, ${\Delta }_{x}\delta {E}_{{\rm{G}}}^{\bigsqcup }$ was characterized by higher degree polynomials undergoing the nonlinear stress fields in the flexural and longitudinal modes outside the core region (see Figs. 2(a) and 3(a)). The combined effects of the hydrostatic and shear stresses experienced by a direct bandgap semiconductor QD can be described by the Bir-Pikus Hamiltonian^50^

1 $${E}_{{\rm{G}}}={E}_{{\rm{H}}}+\sqrt{{Q}_{\varepsilon }^{2}+{R}_{\varepsilon }^{2}+{S}_{\varepsilon }^{2}},$$

where the hydrostatic and shear deformation energies, *E*_*H*_ and *Q*_*ε*_, *R*_*ε*_, and S_*ε*_, are defined, respectively, as *E*_*H*_ = *a* Tr(*ε*), ${Q}_{\varepsilon }=-\,\frac{b}{2}$[Tr(*ε*) − 3*ε*_*zz*_], ${R}_{\varepsilon }=\frac{\sqrt{3}b}{2}({\varepsilon }_{xx}-{\varepsilon }_{yy})-id{\varepsilon }_{xy}$, and ${S}_{\varepsilon }=-\,d({\varepsilon }_{xz}-i{\varepsilon }_{yz})$. Here, *ε*_*ij*_ denotes the components of the strain tensor. The coefficient *a* denotes the hydrostatic deformation potential, whereas the coefficients *b* and *d* denote the valence-band shear deformation potentials, corresponding to the strain tensors with symmetries Γ_1_, Γ_3_, and Γ_4_ ^50^. For the bulk case of GaAs, the shear deformation energy is simply given by *Q*_*ε*_ based on an external uniaxial stress applied along the [001] growth direction. The simple case of strain correspondingly induces the bandgap energy shift $\delta {E}_{{\rm{G}}}=\delta {E}_{{\rm{H}}}+\delta {Q}_{\varepsilon }$ (see Fig. 3(a) and symbols). In practice, under the stress applied by the fundamental flexural oscillation (cf. Fig. 2), various types of shear deformation potentials produce a two-fold decrease in the rate of bandgap change ${\Delta }_{x}\delta {E}_{{\rm{G}}}^{\bigsqcup }$ (red lines and closed red circles, in Fig. 3(a,b)). Shear deformation effects in pillar cavities with three different aspect ratios induce non-zero rates of change (between 0.3 and 0.7 meV/μm; Fig. 3(b)) in the radial breathing mode. The size of the bump in the center (|*r*/*R*| < 0.02) of the energy response curve of the flexural modes increases with the increase in axial ratio of the elliptic pillar cavity (Fig. 3(c)). At an axial ratio of 0.5, the slope of the least-squares-fitted line ${\Delta }_{x}\delta {E}_{{\rm{G}}}^{\bigsqcup }$ for the first flexural mode was twice that of the symmetric cavity resonator. As the quadratic behaviors of the shear deformations of the bump, weakens in the second flexural mode, Fig. 3(c) (blue rectangles) simply depicts the cross-sectional rate of bandgap change, $\delta {E}_{{\rm{G}}}=\delta {E}_{{\rm{H}}}+\delta {Q}_{\varepsilon }$. The cross-sectional rate of bandgap change was simply reduced by two folds.

The quantum confinement potential of an electron–hole system provides a significant correction to the stress-induced exciton energy change. The exciton recombination energy *E*_*X*_ is the sum of the GaAs bulk bandgap *E*_*G*_, the electron–hole sub-band energies *E*_*S*_ of carriers, and the electron–hole Coulomb interaction *J*^51^: ${E}_{{\rm{X}}}={E}_{{\rm{G}}}+{E}_{{\rm{S}}}+J.$ We calculated the quantum mechanical characteristics of the 3D lens-shaped GaAs QDs using an envelope-function method developed within k $\cdot$ p theory (see theoretical details in Methods). For GaAs/Al_0.3_Ga_0.7_As QDs of radius ${R}_{x/y}^{{\rm{QD}}}$ = 7 nm and height *h* = 5 nm, the quantum mechanical correction to the excitonic energy shift $\delta {E}_{{\rm{Q}}}=\delta {E}_{{\rm{S}}}-\delta |J|$ was approximately 70 μeV under a uniaxial hydrostatic stress of 1 MPa (Fig. 3(d)). The substantial correction to the quantum confinement surpasses the stress-dependent bandgap change (60 μeV/MPa; Figs. 2 and 3). The geometrical parameters were obtained from the cross-sectional transmission electron microscope (TEM) images^15,52^. Here, the QD emission energy (1.653 eV) was engineered to couple to the cavity’s resonant mode. Figure 3(d) demonstrates the considerable quantum confinement effects by precisely controlling the ellipticity ${R}_{y}^{{\rm{QD}}}/{R}_{x}^{{\rm{QD}}}$ of pure GaAs QDs. The stress-induced confinement characteristics, ${\Delta }_{{\rm{S}}}{E}_{{\rm{Q}}}^{\bigsqcup }$, were determined by the slope of a quantum confinement energy–stress curve obtained using a 3D envelope-function formalism. We evaluated the 3D QD confining potentials relative to structural parameters (${R}_{x/y}^{{\rm{QD}}}$ and *h*) and the uniaxial stress-induced change in the confinement geometry. At a minor-major axis ratio of ~0.9, the stress-induced rate ${\Delta }_{{\rm{S}}}{E}_{{\rm{Q}}}^{\bigsqcup }$ was changed by 10% (7 μeV/MPa) relative to the symmetric QD. The quantum-confinement effect $\delta {E}_{{\rm{Q}}}^{\bigsqcup }$ has a standard deviation of 3% over an axial ratio of 0.5–1.

Furthermore, we investigated the effect of Al interdiffusion on the excitonic band gap in the GaAs DE QDs. To construct a realistic confinement potential profile, we studied the empirical in-depth Al profile for an optimized type of GaAs DE QD^15,52^. In a previous two-dimensional study, we measured the position-dependent mole fraction *x*(*z*) of Al along the QD’s growth axis using TEM-energy dispersive X-ray spectroscopy. Then, we applied the polynomials obtained via least-squares-fitting to the well-known bandgap formula ${E}_{{{\rm{Al}}}_{{\rm{x}}}{{\rm{Ga}}}_{(1-{\rm{x}})}{\rm{As}}}(z)$ = 1.519 + 1.155 *x*(*z*) + 0.37 *x*(*z*)^2^ ^53^. According to this analysis wherein we used 3D envelope-function modeling, the DE QD recombination energy red-shifted by ~1.7% and the quantum confinement correction of ~16%. In addition, the stress-induced shift rate ${\Delta }_{{\rm{S}}}{E}_{{\rm{Q}}}^{\bigsqcup }$ changed by 5% in its realistic confining potential profile of Al-interdiffused QDs (cf. Fig. 3(e)). Our 3D calculation showed that the individual rate of change ${\Delta }_{{\rm{S}}}{E}_{{\rm{Q}}}^{\bigsqcup }$, which depended on the shape anisotropies of the confinement potential in the GaAs QDs, was also shifted by approximately 5%. These results provide the practical margins for GaAs QD tuning and for improving the source performances of QD SPS applications (such as nondestructive position mapping and imaging methods). Our methodology presented in this study can be directly implemented in versatile micropillar–microcavity embedding quantum emitters. For quantum telecommunication on optical fiber networks, such precise control can be utilized in hybrid two-level systems such as InGaAsP/InP^54–56^ and GaSb/GaAs QDs^57,58^, clad by lattice-matched DBR pillars. In general, our methods can be applied to improve the source performance of different types of solid-state quantum-emitters embedded in cylindrical structures. Because the quantum error in the energy shift was estimated down to $\mathrm{5 \% }{E}_{{\rm{Q}}}^{{\rm{Al}}}/{E}_{{\rm{X}}}$, the subwavelength imaging/positioning of QDs theoretically outperforms other optical nanoscopy such as the stimulated emission depletion, in which 5-nm resolution of nitrogen-vacancy centers was achieved^59^. Meanwhile, the practical quantum error is set by employing the quantum-mechanical corrections in the individual confinement geometries of solid-state emitters. Destructive composition depth-profile analyses of individual emitters are mainly needed for their high-precision control at the quantum level.

## Conclusion

We have theoretically demonstrated full frequency control of strain-free GaAs QDs embedded in micropillar cavity SPSs. By harnessing strain coupling, we fine-tuned the QD excitonic energy with cross-sectional shift rates of few meV/μm in tailored micropillar optomechanical resonators. Using an envelope-function model of 3D GaAs QDs, we demonstrated that the tuning/positioning precision of the quantum confinement energy is limited to 5% by varying shapes and compositions of the QDs. Our approach, which exploits the nano-optomechanics of QD-cavity systems, is fully compatible with integrated photonic circuits, providing an intriguing avenue for developing hybrid quantum and classical computers.

## Methods

We employ a 3D envelope-function method developed within k $\cdot$ p perturbation theory to calculate the quantum mechanical characteristics of lens-shaped DE QDs of GaAs/Al_0.3_Ga_0.7_As. The geometrical parameters such as height and base lengths were extracted from the cross-sectional TEM images^15,52^. The QD energy of a single electron–hole pair comprises the GaAs bulk bandgap *E*_*G*_, the sub-band energies *E*_*S*_ of the carriers, and the direct Coulomb interaction energy *J*. Here, the quantity ${E}_{{\rm{Q}}}={E}_{{\rm{S}}}-|J|$ characterizes the quantum confinement of an electron–hole system in the QD. To determine the sub-band confinement energies *E*_*S*_ and the electron–hole Coulomb interaction energy *J* in the stress-dependent QD confinement potential *V*(**r**), we numerically solved the following Schrödinger equation:

$$-\frac{{\hslash }^{2}}{2{m}^{\ast }({\bf{r}})}\frac{{\partial }^{2}}{{\partial }^{2}{\bf{r}}}\psi ({\bf{r}})+V({\bf{r}})\psi ({\bf{r}})=E\psi ({\bf{r}}\mathrm{)}.$$

Using a finite-difference method, we evaluated the 3D Coulomb integral of *J* projected onto the exciton state |*ψ*_*e*_*ψ*_*h*_〉 defined as ^15,51^ $J=-\,{e}^{2}{\sum }_{\sigma ,\sigma ^{\prime} }\int \int {d}^{3}r^{\prime} {d}^{3}r\times \frac{\psi \ast e({\bf{r}},\sigma ){\psi }_{e}({\bf{r}},\sigma )\psi \ast h({\bf{r}}{\boldsymbol{^{\prime} }},\sigma ^{\prime} ){\psi }_{h}({\bf{r}}{\boldsymbol{^{\prime} }},\sigma ^{\prime} )}{\varepsilon ({\bf{r}},{\bf{r}}{\boldsymbol{^{\prime} }})|{\bf{r}}-{\bf{r}}{\boldsymbol{^{\prime} }}|}$, for the dielectric screening *ε*(**r**, **r**′) at a given position *r* with spin *σ*. The material parameters used in the calculation are given elsewhere^15,60^.
